# Dietary supplementation ellagic acid on the growth, intestinal immune response, microbiota, and inflammation in weaned piglets

**DOI:** 10.3389/fvets.2022.980271

**Published:** 2022-09-02

**Authors:** Yujie Lu, Mingwei Zhao, Jiayuan Mo, Ganqiu Lan, Jing Liang

**Affiliations:** ^1^College of Animal Science and Technology, Guangxi University, Nanning, China; ^2^Guangxi Guiken Jinmao Animal Husbandry Co., Ltd., Nanning, China

**Keywords:** ellagic acid, intestinal mucosa, immune response, inflammation, pig

## Abstract

Piglets are susceptible to weaning stress, which weakens the barrier and immune function of the intestinal mucosa, causes inflammation, and ultimately affects animal growth and development. Ellagic acid (EA) is a natural polyphenol dilactone with various biological functions. However, The mechanisms underlying the effects of EA on animal health are still poorly known. Herein, we examined whether dietary supplementation with EA has a positive effect on growth performance, intestinal health, immune response, microbiota, or inflammation in weaned piglets. Sixty weaned piglets (age, 30 days) were randomly divided into two groups: the control group (basic diet) and the test group (basic diet + 500 g/t EA). The pigs were fed for 40 days under the same feeding and management conditions, and the growth performance of each individual was measured. At the end of the feeding period, samples were collected from the small intestinal mucosa for further analysis. Using these tissues, the transcriptome sequences and intestinal microbial diversity were analyzed in both groups. An inflammation model using small intestinal mucosal epithelial cells (IPEC-J2) was also constructed. Dietary EA supplementation significantly increased the average daily weight gain (ADG) and reduced diarrhea rate and serum diamine oxidase (DAO) levels of weaned piglets. Transcriptome sequencing results revealed 401 differentially expressed genes in the jejunum mucosal tissue of pigs in the control and test groups. Of these, 163 genes were up-regulated and 238 were down-regulated. The down-regulated genes were significantly enriched in 10 pathways (false discovery rate < 0.05), including seven pathways related to immune response. The results of bacterial 16s rDNA sequencing show that EA affects the composition of the intestinal microbiota in the cecum and rectum, and reveal significant differences in the abundances of *Prevotella_9, Lactobacillus delbrueckii*, and *Lactobacillus reuteri* between the test and control groups (*P* < 0.05). Experiments using the inflammation model showed that certain doses of EA promote the proliferation of IPEC-J2 cells, increase the relative mRNA expression levels of tight junction-related proteins (*ZO-1* and *Occludin*), improve the compactness of the intestine, reduce the expression of inflammatory factors *TNF-*α and *IL*-6, and significantly reduce LPS-induced inflammation in IPEC-J2 cells. In conclusion, we found for the first time that dietary supplementation of EA affects the gut immune response and promotes the beneficial gut microbiota in weaned piglets, reduces the occurrence of inflammatory responses, and thereby promotes the growth and intestinal health of piglets.

## Introduction

The gut is the body part most vulnerable to harmful molecular stress. However, it is also the largest immune organ in animals, and ~70% of all immune cells in the body are derived from the gut (1). The intestinal immune barrier has various functions and can stimulate the body to produce an immune response to a processed antigen. As an independent immune system, the intestinal immune barrier is composed of lymphocytes, immune cells, and cytokines (2). Gut microbes play a crucial role in maintaining host health; for example, gut microbes affect nutrient absorption, feed digestion, and energy supply, and regulate physiological functions and the occurrence and development of diseases (3). In piglets, weaning can cause stress, change the intestinal morphology, and damage the intestinal mechanical barrier, thereby causing immune and inflammatory reactions and even death (4). In the past time of the pig industry, the usual response to this stress was to add antibiotics to the diet; however, the abuse of antibiotics has caused many problems. Since 2020, China has instituted a comprehensive ban on the use of antibiotics in the pig feed industry, and finding effective substitutes for antibiotics has become an urgent task. Several studies have reported that enzyme preparations, probiotics, and plant extracts can be used as substitutes for antibiotics. As such, numerous studies have shown that ellagic acid (EA)—a natural plant polyphenol that is widely present in various plant tissues—has excellent antimutation, antibacterial, anti-cancer, anti-inflammatory, and antioxidant functions (5).

EA and its derivatives have an inhibitory effect on the oxidation of microsomes and mitochondria in mammalian cells and play a role in delaying aging. EA also inhibits inflammation in the ileum of mice (6) and its derivatives have obvious inhibitory effects on chemically-induced and other types of carcinogenesis (5). In particular, EA has been shown to effectively reduce paraquat-induced hepatic oxidative damage and inflammation by modulating the cecal microbial community, providing a new nutrition-based therapeutic strategy for liver injury (7). A study by Li et al. suggested that EA may be used as a natural antifungal drug to treat diseases (8). Moreover, EA has been shown to prevent wound infection and ulceration by inhibiting bacteria *in vitro*.

Specific doses of EA have been shown to increase the weight of normal mice. Moreover, EA alleviates the symptoms of colitis and even has a therapeutic effect in a mouse model of colitis (9). These findings indicate that EA has anti-inflammatory effects and positively influences animal growth. However, few studies have shown the influence of EA on inflammation in weaned piglets or its mechanism of action. To investigate this, we performed transcriptome sequencing and 16S rDNA analysis and constructed an inflammatory model for verification. In this study, we explored whether EA could reduce the level of inflammation in weaned piglets by affecting the gene expression patterns and microbiota composition of the pig intestine, and aimed to improve the understanding of the mode of action of EA as a feed additive and facilitate the application of new technologies.

## Materials and methods

### Experimental animals and sample collection

We purchased 60 healthy piglets (30-day-old; 10 ± 0.51 kg; Landrace × Yorkshire, barrow) from Guangxi State Farms Yongxin Livestock Husbandry Group Co., Ltd. (China). The piglets were randomly divided into a test group (30 pigs, 10 pigs/pen) and a control group (30 pigs, 10 pigs/pen). EA (90%) was purchased from Xiangtan Jiayeyuan Biotechnology Co., Ltd. (Hunan, China). The piglets were raised in the Experimental Base of Guangxi University and provided the same formula feed twice daily at 8:00 and 16:00. The basal diet contained 58% quality corn, 25% dehulled soybean meal, 18% crude protein, and 3,000 kJ digestible energy (Supplementary Table 1); however, EA (500 g/t) was added to the diet of the test group. Water was available *ad libitum*, and the experimental period was 40 days. All piglets were managed in accordance with the routine management of the pig farm. The feces of the piglets were monitored and examined daily. To determine growth performance, initial weight (day 0 of feeding) and final weight (day 40 of feeding) were measured for each piglet and the ADG of each individual was calculated. At the end of the experimental period, blood samples were collected from the anterior vena cava to measure DAO activity in serum. One pig was randomly selected for slaughter from each pen of the control group and the test group (six pigs in total). The piglets were were fasted for 12 h before slaughter. Tissue and mucosal samples were collected from the ileum and jejunum and sectioned for intestinal morphological observation, and the contents of the jejunum, ileum, cecum, and rectum were sampled. All samples were immediately frozen in liquid nitrogen and stored at −80 °C until use.

### Transcriptome analysis of the jejunal mucosal tissue

Total RNA was extracted from the jejunal tissue using Trizol (Ambion, Life Technologies, USA). The concentration and purity of the total RNA were measured with a microspectrophotometer (Quawell, USA) to ensure that the quality of the total RNA met the test requirements. mRNA was isolated from the total RNA samples using oligo (DT) magnetic beads. The purified mRNA was fragmented with a fragmentation buffer and used as a template to synthesize two strands of cDNA. Following this, a one paired-end library was prepared for each sample. After the library was qualified, PE150 paired-end sequencing was performed using the Illumina sequencing platform.

The raw data from the Illumina sequencing platform were quality controlled and filtered (FASTX Toolkit v0.0.13), and the sequencing joints, low-quality sequences, and duplicates were removed. The clean reads were assembled, and the available sequences were obtained by removing redundant sequences and splicing and clustering homologous transcripts. The filtered sequences were compared to the *Sus scrofa* reference genome (v11.1), and the mRNA expression levels were calculated and analyzed. The fragments per kilobase per million mapped fragments (FPKM) index was used to measure differences in gene expression between different samples, and was calculated as follows:


$$\begin{array}{l} FPKM=\frac{10^{3}*F}{NL/10^{6}} \end{array}$$

where F is the number of fragments uniquely matched to this gene, N is the total number of fragments compared to the reference gene, and L is the length of the exon region of the gene.

The differentially expressed genes between groups were screened and analyzed by Gene Ontology (GO) and Kyoto Encyclopedia of Genes and Genomes (KEGG) enrichment analysis. The corresponding GO identifiers and annotation terms were mainly found using the gene ID or sequence annotation.

To validate the veracity and reliability of the transcriptome data, seven genes were randomly selected for RT-qPCR validation. The RT-qPCR of the target genes was performed using the ABI 7,500 real-time PCR system (Applied Biosystems, Carlsbad, CA, USA) and ChamQ Universal SYBR qPCR Master Mix (Vazyme Biotech Co., Ltd., Q711-02/03, Nanjing, China). All primers were synthesized by Sangon Biotech Co., Ltd. (Shanghai, China) with the sequences shown in Supplementary Table 2. The relative quantification of the gene was calculated using the formula 2^−Δ*ΔCt*^ method with 18S as the reference gene.

### 16S RDNA sequencing of the intestinal contents of piglets

The MagPure Stool DNA LQ Kit (Magen, D6358-03, China) was used to extract total bacterial DNA from the fecal samples according to the manufacturer's instructions. The V3–V4 region of the 16S rRNA gene was amplified with the primers 341F (5-CCTACGGGNGGCWGCAG-3) and 806R (5-GGACTACHVGGGTATCTAAT-3). The PCR products were used for library construction and sequenced using MiSeq-PE250 sequencing on an Illumina Hiseq 2500 platform. The reads at both ends were spliced into units with a minimum length of 10 bp through FLASH to obtain raw reads. According to the QIIME (v1.9.1) tag quality control process, the raw tags were truncated from the first site with consecutive low-quality bases (default quality threshold: ≤ 3) until it reached the set length (default length = 3). The tags were further filtered to remove tags whose consecutive high-quality bases comprised <75% of the total length and tags shorter than 300 bp. The filtered sequences were compared using homologous clusters to obtain operational taxonomic units (OTUs). The α and β diversity indices were measured and analyzed. Linear discriminant analysis of effect size (LEfSe) was used to identify the differential microbiota, and PICRUSt analysis was used to predict microbial functions.

### Construction of an inflammatory model of small intestinal mucosal epithelial cells

IPEC-J2 cells were donated by the College of Animal Sciences, Zhejiang University (Zhejiang, China). The cells were maintained in DMEM medium (Life Technologies USA) containing 10% (v/v) fetal bovine serum (Corning, Inc., USA) and 1% double antibody (Thermo Fisher Scientific, USA), and incubated at 37 °C and 70–80% humidity with 95% oxygen and 5% CO_2_ (v/v). The growth of IPEC-J2 cells was observed under a microscope. When the cells had grown to 80–90% of the area of the culture plate, we performed cell passage, monitoring the cell status every 12 h.

The experiment consisted of five parts as follows:

(I) Effect of EA on the viability of IPEC-J2 cells. The treatments included a negative control, blank, and treatments with 10, 20, 50, and 100 μg/mL EA. The treatments were repeated three times in three 96-well-plates (Corning, Inc.), and the plates were placed in an incubator (Thermo Fisher Scientific, USA) and cultured for 6, 12, and 24 h, respectively. After culturing, 50 μL MTT (Abcam, China) was added, and the plates were further cultured for 4 h. Subsequently, 150 μL dimethyl sulfoxide (Thermo Fisher Scientific, USA) was added to each hole of the 96-well-plates, and the cultures were incubated for 15 min. Absorbance was measured at 490 nm using a microplate reader.

(II) Effect of lipopolysaccharides (LPS) on the viability of IPEC-J2 cells. The treatments included the negative control, blank, and treatments with 0.1, 1, and 10 μg/mL LPS (*Escherichia coli* serotype 0111:B4, Sigma-Aldrich, USA). The treatments were repeated three times, and the plates were cultured for 12, 24, and 48 h. The remaining steps were the same as in (I).

(III) Effect of EA on the activity of IPEC-J2 cells induced by LPS. The four treatments included the control and treatments with 50 μg/mL EA, 10 μg/mL LPS, and 50 μg/mL EA + 10 μg/mL LPS. The remaining steps were the same as in (I).

(IV) Effect of EA on tight junction-related genes in IPEC-J2 cells. RT-PCR was used to detect the gene expression of target genes, including *Occludin, TNF-*α, *ZO-1*, and *IL-6*. The primers were designed using Oligo 7 primer analysis software (Molecular Biology Insights) (Supplementary Table 3).

(V) Effect of EA on the expression of inflammatory factors in IPEC-J2 cells induced by LPS. IPEC-J2 cells were cultured in 6-well-plates in two treatment groups—control and 10 μg/mL LPS—and used to analyze the expression of inflammatory factors by RT-PCR.

### Statistical analysis

All data were analyzed using SPSS software (SPSS Statistics 20, IBM Japan, Ltd., Tokyo, Japan). The values of the diversity indices and richness indices were tested by the Student's *t-*test. The absorbance of the IPEC cells was analyzed by one-way ANOVA. Data was presented as the mean ± SEM. Differences were considered significant when *P* < 0.05.

## Results

### Effects of EA on growth performance and intestinal barrier function of weaned piglets

The initial weight was not significantly different between the control (without EA) and test (with EA) groups (*P* > 0.05). The final weight and ADG were significantly higher among piglets in the test group than among those in the control group (*P* < 0.05), and the diarrhea rate was lower in the test group than in the control group (*P* < 0.05). The serum DAO levels were significantly higher in the control group than in the test group (*P* < 0.05) (Supplementary Table 4) (10).

### Processing and analysis of transcriptome data

After quality control, we obtained a total of 27.9 G of clean reads for six samples. The clean reads of each sample ranged in size from 4.4 G to 4.7 G. For the CK01, CK02, and CK03 samples, a total of 95.43, 95.98, and 95.79% of clean reads aligned to the reference genome, respectively. For the EA01, EA02, and EA03 samples, 95.71, 95.90, and 95.80% of the clean reads aligned to the reference genome, respectively. This indicates that the transcriptome data was accurate and reliable.

### Differentially expressed genes between the test and control groups

**Figure 1:**
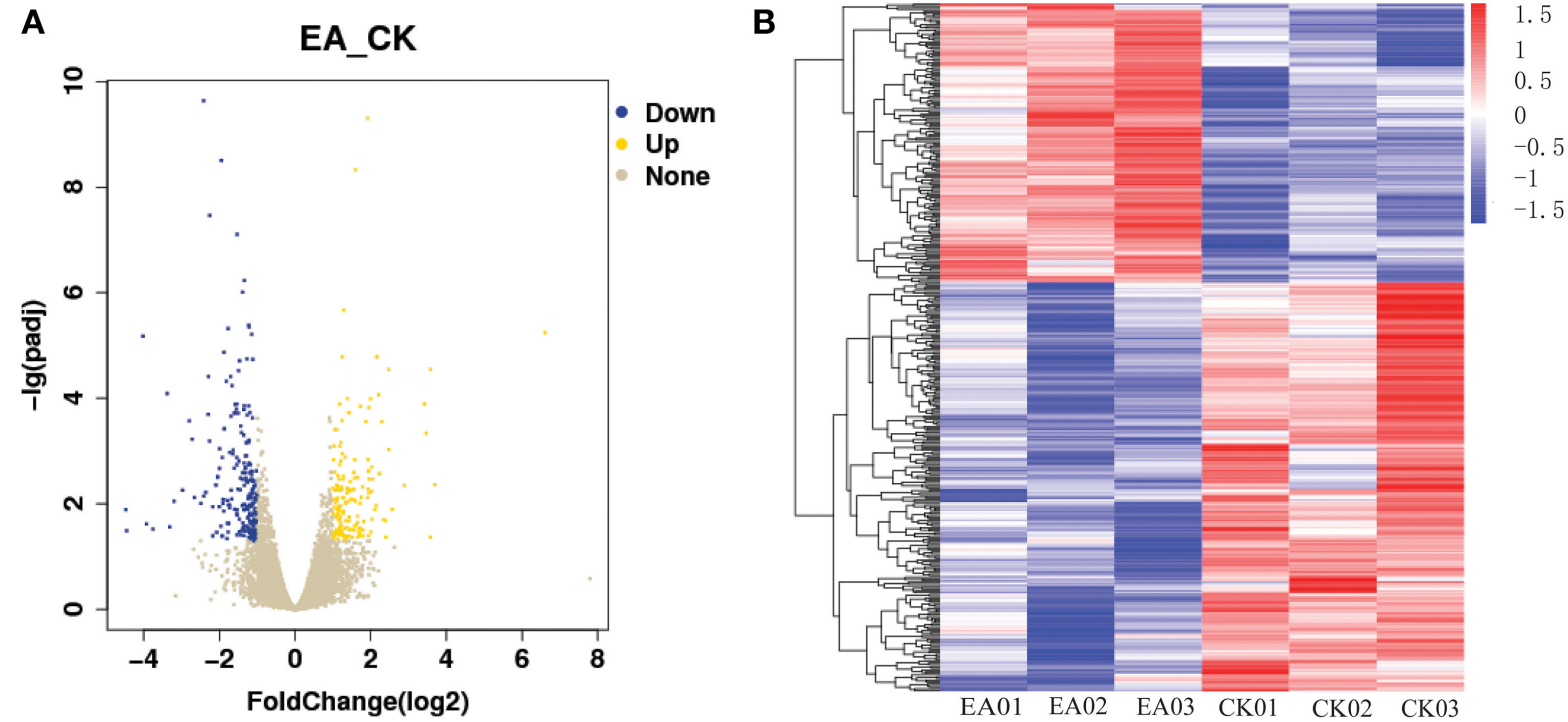
The differentially expressed gene map. **(A)** Is the volcano map of differentially expressed gene, **(B)** is the heatmap of differentially expressed gene.

The TopHat software (Center for Computational Biology at Johns Hopkins University) and FPKM algorithm were used to calculate the expression levels of genes in the different groups of samples. There was a total of 17,700 jejunum mucosal genes in the control and test groups, and most of the genes were common between the groups. A total of 401 genes were significantly differentially expressed between the groups (q-value < 0.05), including 163 up-regulated and 238 down-regulated genes (Figure 1). GO analysis and KEGG enrichment analysis showed that the 238 down-regulated genes were significantly enriched in 10 pathways (false discovery rate < 0.05), of which 7 were related to immune response. These included the T cell co-stimulation (GO:0031295), immune response (GO:0006955), immunological synapse (GO:0001772), cytokine-cytokine receptor interaction (ssc04060), T cell receptor signaling (ssc04660), NK cell mediated cytotoxicity (ssc04650), and primary immunodeficiency (ssc05340) pathways. The genes significantly enriched in these pathways included *CCL*5, *CCL*20, *CXCL*11, *CXCL*16, *IL*2*RB, IL*2*RG, IL*9*R, IL*21*R, CD*5, *CD*3*E, CD*244, and *GZMB*, among others (Table 1).

**Table 1:** The GO and KEGG pathways of RNA-Seq.

**Category**	**Term**	**FDR**	**Count**	**Genes**
GOTERM_BP_DIRECT	GO:0031295~T cell costimulation	0.0034	5	SPN, TMIGD2, TNFSF14, CD5, CD3E
GOTERM_BP_DIRECT	GO:0006955~immune response	0.0034	12	CXCL11, TMIGD2, TNFSF14, OAS2, CCL20, CCL5, TNFRSF9, GZMB, XCL1, B2M, LAT, CD244
GOTERM_CC_DIRECT	GO:0009897~external side of plasma membrane	0.0060	9	SPN, CD200R1, CD8B, CD8A, CD5, TNFRSF9, CD69, CD3E, CD244
GOTERM_CC_DIRECT	GO:0001772~immunological synapse	0.0072	5	LCK, CD37, CD3E, CORO1A, LAT
KEGG_PATHWAY	ssc04060:Cytokine-cytokine receptor interaction	0.0238	11	CXCL11, TNFSF14, CCL20, CCL5, TNFRSF9, IL2RB, IL21R, ACKR3, IL2RG, IL9R, CXCL16
KEGG_PATHWAY	ssc04660:T cell receptor signaling pathway	0.0238	8	CD8B, CD8A, LCK, FYN, CD247, FOS, CD3E, LAT
KEGG_PATHWAY	ssc04650:Natural killer cell mediated cytotoxicity	0.0238	8	LCK, SH2D1B, PRF1, GZMB, FYN, CD247, LAT, CD244
KEGG_PATHWAY	ssc05340:Primary immunodeficiency	0.0451	5	CD8B, CD8A, LCK, IL2RG, CD3E
GOTERM_BP_DIRECT	GO:0008360~regulation of cell shape	0.0481	7	FGR, SEMA4D, SH3KBP1, WIPF3, PLEKHO1, FYN, CORO1A
GOTERM_BP_DIRECT	GO:0042127~regulation of cell proliferation	0.0497	8	FGR, CNN2, CXCL11, NOS2, LCK, TNFRSF9, FYN, SGK1

### Validation of RNA-Seq results by RT-QPCR

**Figure 2:**
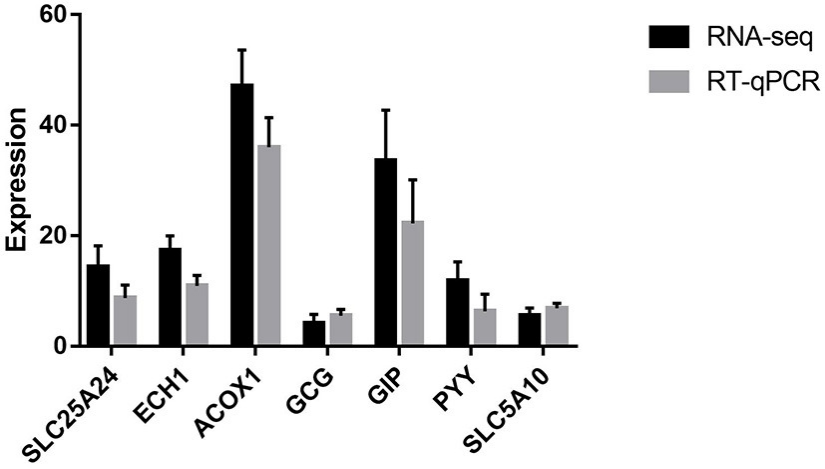
RT-qPCR validation of the selected seven genes. *SLC25A24* solute carrier family 25 member 24; *ECH1*, enoyl-CoA hydratase 1; *ACOX1*, acyl-CoA oxidase 1; *GCG*, glucagon; *GIP*, gastric inhibitory polypeptide; *PYY*, peptide YY; *SLC5A10*, solute carrier family 5 member 10.

To validate the transcriptomic results by RT-qPCR, seven genes (*ACOX*1, *ECH*1, *GCG, GIP, PYY, SLC*25*A*24, and *SLC*5*A*10) were validated. As shown in Figure 2, the expression profiles of these genes detected by RT-qPCR were consistent with those detected by transcriptome, which confirmed the reliability of our RNA sequencing data.

### Comparison of the diversity and composition of intestinal microbiota between the test and control groups

**Figure 3:**
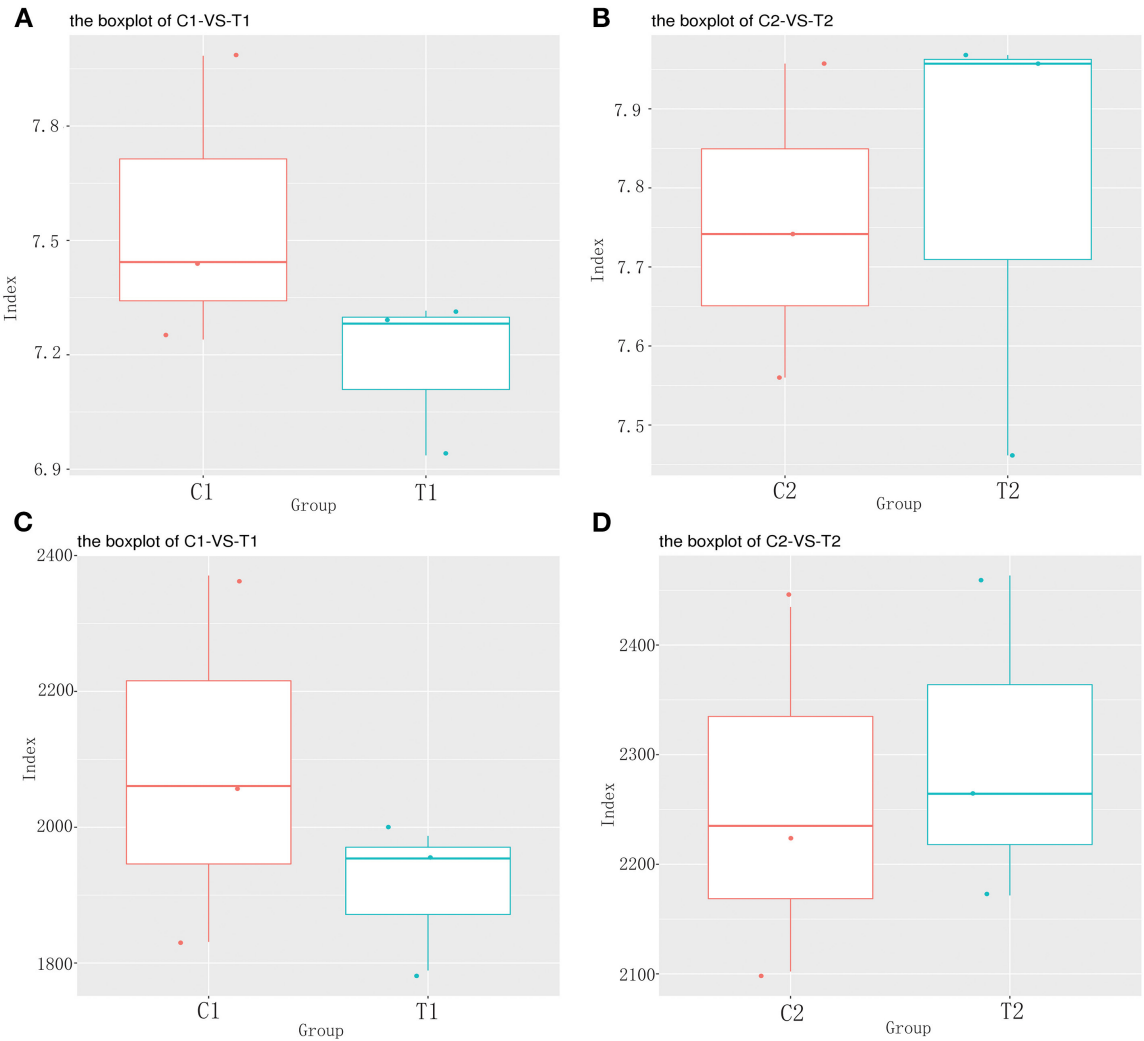
The α diversity analysis results. **(A)** Cecum Shannon index; **(B)** Rectum Shannon index; **(C)** Cecum Chao 1 index; **(D)** Rectum Chao 1 index.

**Figure 4:**
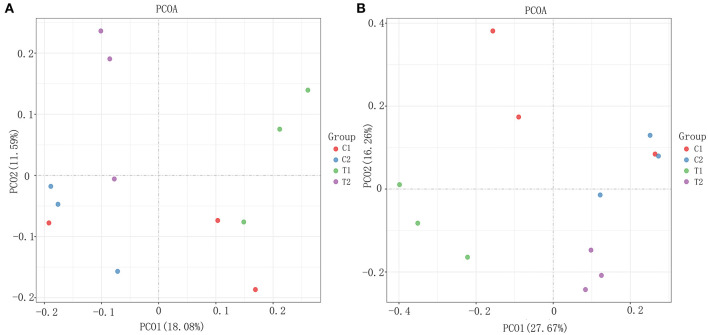
The β diversity analysis results. **(A)** Weighted Unifrac PCoA scatter plot; **(B)** Jaccard PCoA scatter plot.

To evaluate the effect of EA on the intestinal microbiota, 12 samples of the cecal and rectal contents were analyzed. 16S rDNA gene sequencing was used to analyze changes in the microbial composition of the pig intestine. An average of 83,766 high-efficiency tags per sample were obtained. At 97% pairwise sequence identity, an average of 1,653 OTUs per sample were obtained. α diversity indices (Shannon and Chao 1) were used to evaluate the diversity of intestinal microbiota in the samples from each group. The results of sequence analysis showed a consistent trend of microbial diversity in the two tissue samples (Figure 3). In the cecum, the Shannon and Chao 1 indices were slightly lower in the test group than in the control group (*P* > 0.05) (Figures 3A,C). β diversity indices were also analyzed to identify the differences in intestinal microbial composition between groups by comparing the unweighted UniFrac statistics and Jaccard coefficients (Figure 4). The results showed that the intestinal microbiota of the control and test groups were completely separated, and that the microbiota were widely dispersed among different groups. This shows that EA affects the composition of the intestinal microbiota in the cecum and rectum.

**Figure 5:**
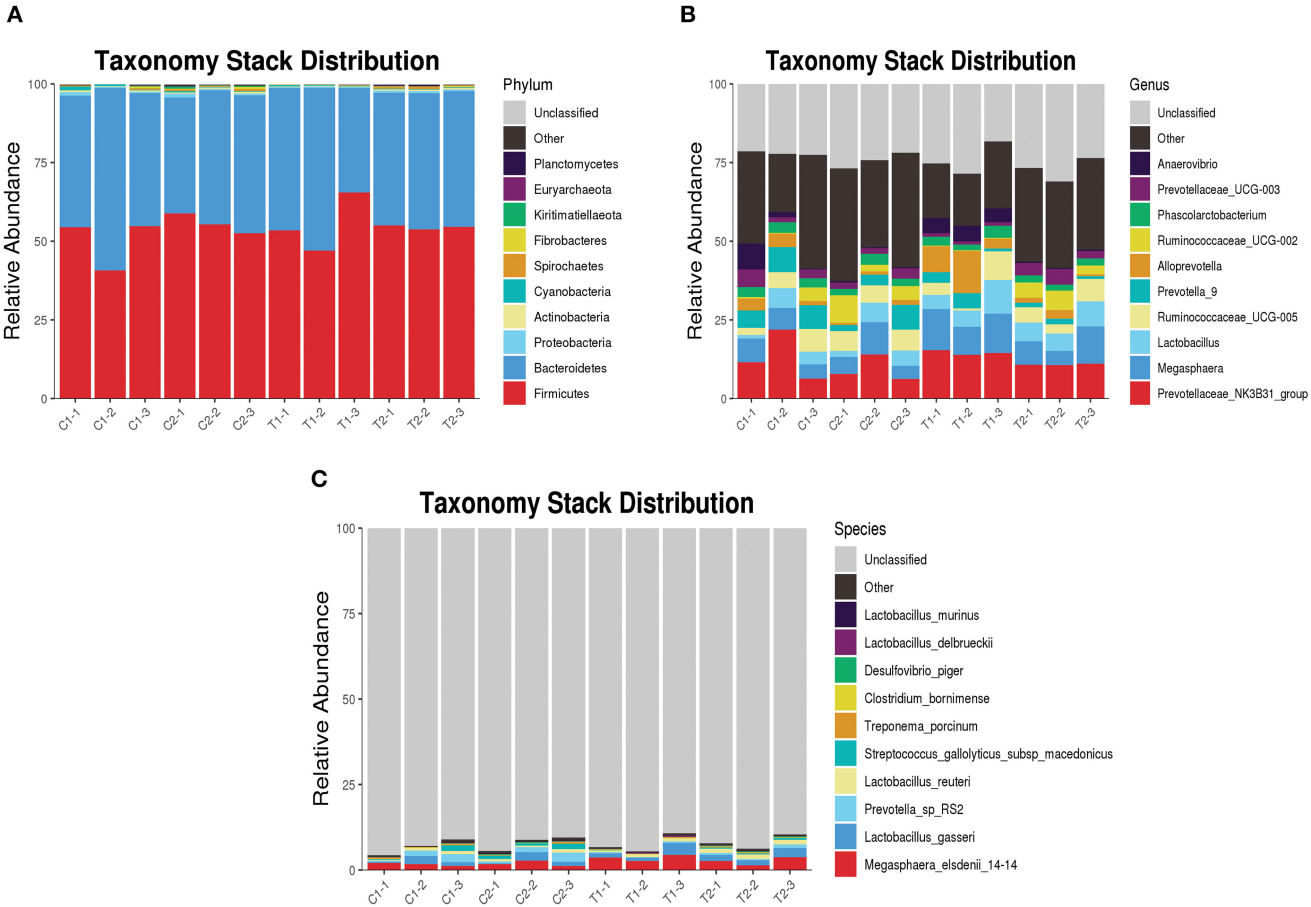
Taxonomy stack distribution of various species. **(A)** Taxonomy stack distribution of phylum level; **(B)** Taxonomy stack distribution of genus level; **(C)** Taxonomy stack distribution of species level.

The relative abundance of bacterial taxa at the phylum, genus, and species levels between the test and control groups were also compared. At the phylum level, Firmicutes and Bacteroidetes were the dominant phyla in the cecum and rectum in both groups, accounting for more than 90% of all microbes (Figure 5A). At the genus level, *Megasphaera* was significantly more abundant in the ceca of animals in the test group than in those of the control group (*P* < 0.05), and the abundance of *Prevotella*_9 was significantly lower in the test group than in the control group (*P* < 0.05) (Figure 5B). At the species level, the abundances of *Lactobacillus delbrueckii* and *Megasphaera_elsdenii_14-14* were significantly higher in the ceca of animals in the test group than in those of the control group (*P* < 0.05); and the abundance of *Lactobacillus_reuteri* was significantly higher in the rectums of animals in the test group than in those of the control group (*P* < 0.05) (Figure 5C).

**Figure 6:**
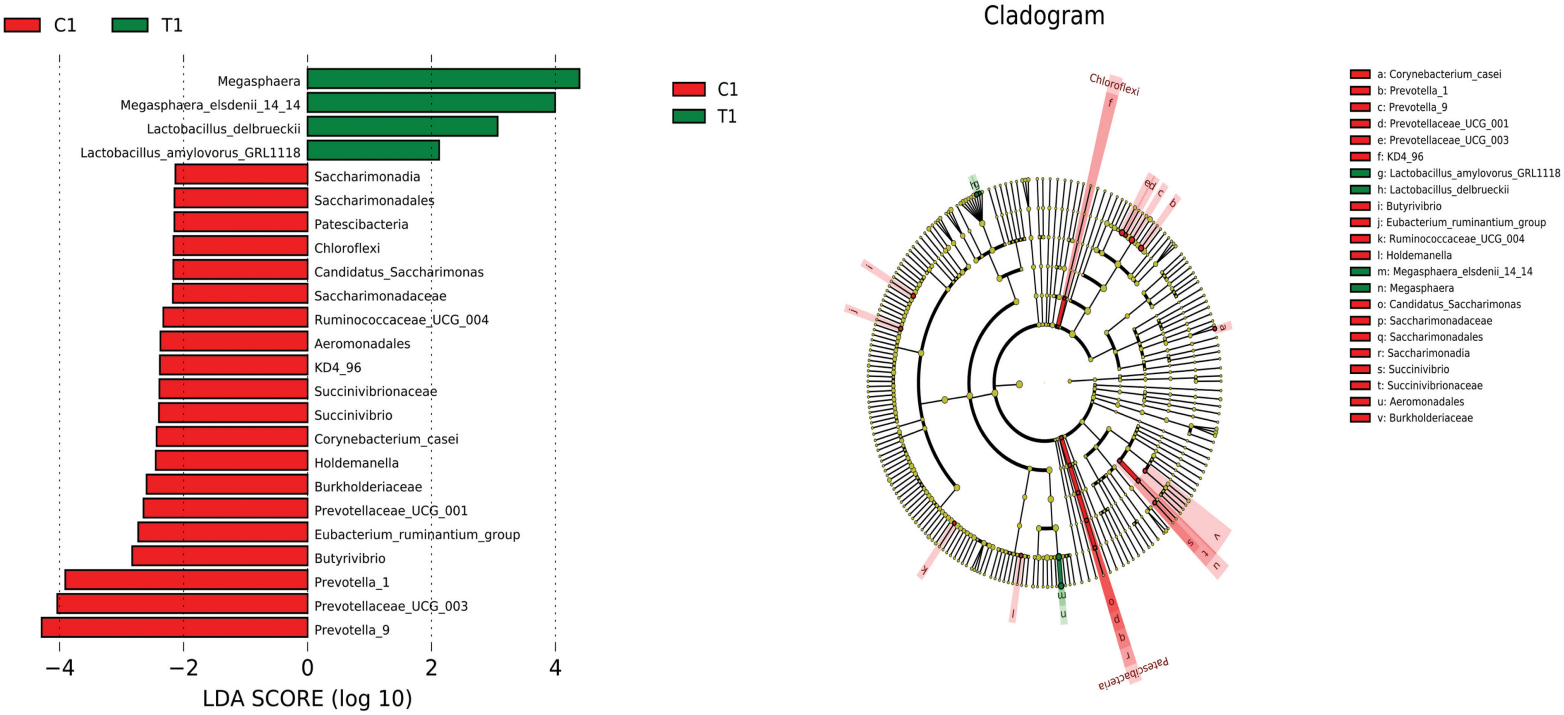
LEfSe analysis diagram of cecum.

**Figure 7:**
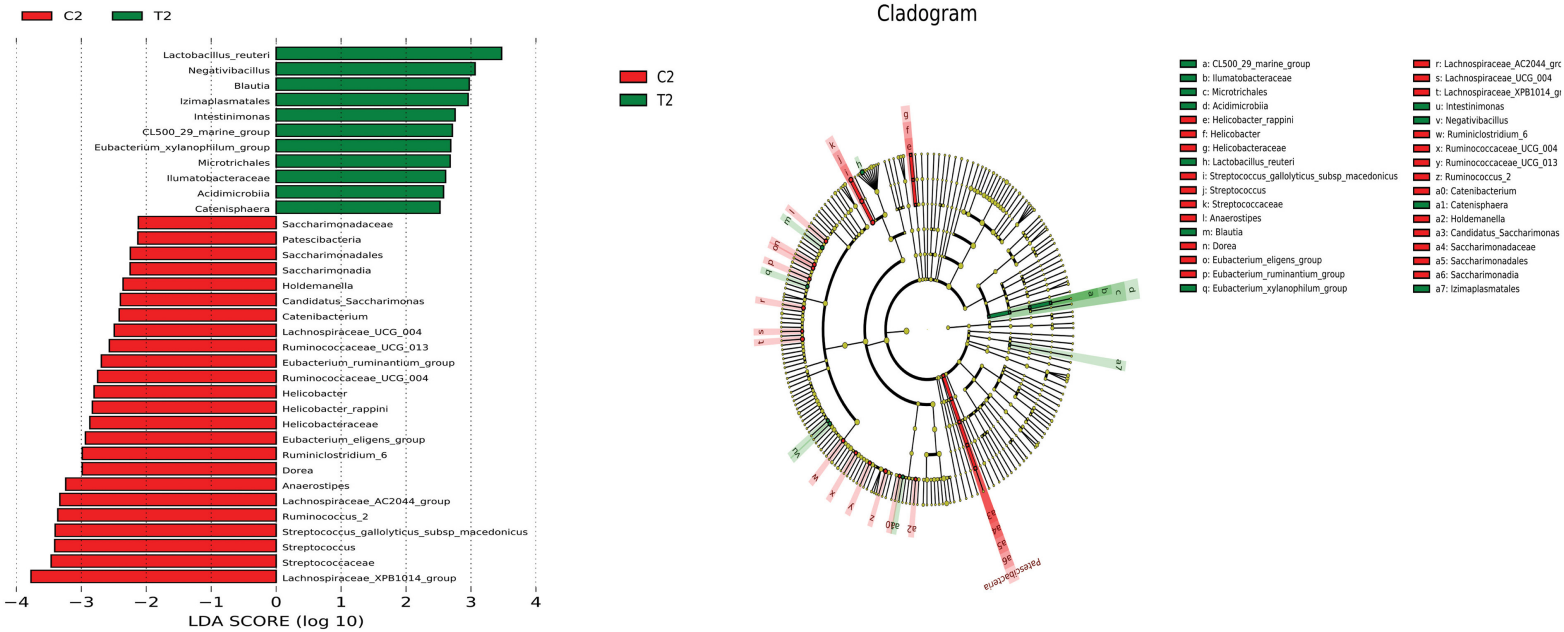
LEfSe analysis diagram of rectum.

LEfSe analysis was used to compare the microbiota in the cecal and rectal contents of the two groups. In the cecum, the abundances of *Megasphaera, Megasphaera elsdenii_14_14, Lactobacillus delbrueckii*, and *Lactobacillus amylovorus* were higher in the test group than in the control group (*P* < 0.05) (Figure 6). In the rectum, the abundances of *Lactobacillus reuteri, nagativibacillus, Blautia, Microtrichales, Acidimicrobiia*, and *Catenisphaera* were higher in the test group than in the control group (*P* < 0.05) (Figure 7).

**Figure 8:**
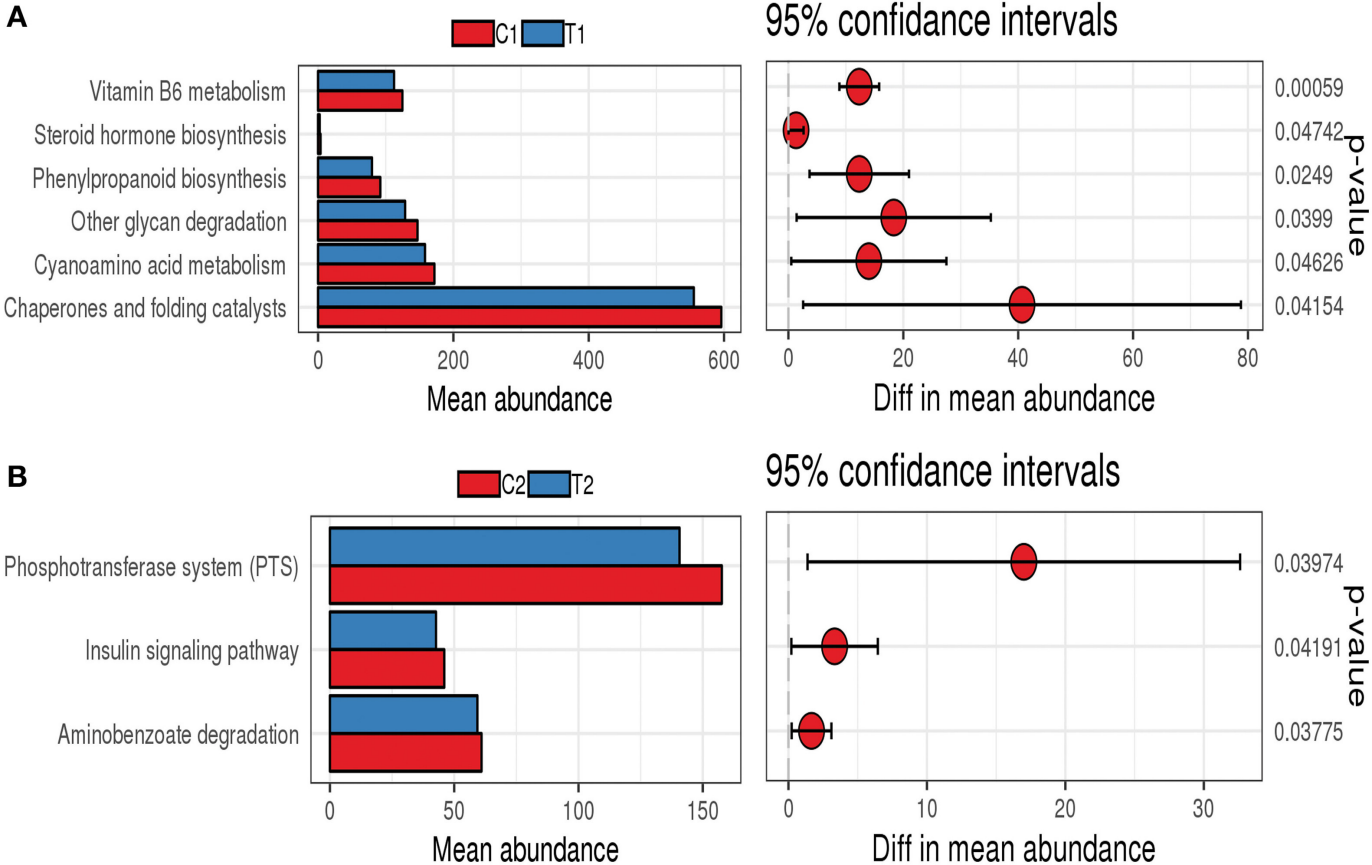
PICRUSt function prediction chart. **(A)** Cecum; **(B)** Rectum.

PICRUSt analysis was used to infer the functions of intestinal microbes in different groups. The functions of the bacterial microbiota in the cecum and rectum differed between the test and control groups. The functions of the bacterial microbiota in the cecal contents of the test group were mainly enriched in vitamin B6 metabolism, cyanoamino acid metabolism, other glycan degradation, phenylpropanoid biosynthesis, and steroid hormone biosynthesis (Figure 8A). In contrast, the functions of the bacterial microbiota in the rectal contents of the test group were mainly enriched in the phosphotransferase system (PTS), aminobenzoate degradation, and the insulin signaling pathway (Figure 8B).

### Effect of EA on inflammation of small intestinal mucosal epithelial cells

#### Effect of EA on the viability of IPEC-J2 cells

Following treatment with EA for 6 h, IPEC-J2 cells treated with 100 μg/mL EA showed significantly higher proliferation than cells in the treated with 0 μg/mL EA (control group; *P* < 0.05). However, there were no significant differences among the other groups (*P* > 0.05). Following treatment with EA for 12 h, IPEC-J2 cells treated with 20, 50, and 100 μg/mL concentrations of EA showed significantly higher proliferation than cells in the control group (*P* < 0.01). Following treatment with EA for 24 h, cells treated with 10 μg/mL EA showed significantly higher proliferation than cells in the control group (*P* < 0.05); moreover, cells treated with 20, 50, and 100 μg/mL EA showed even more significantly higher proliferation than cells in the control group (*P* < 0.01) (Table 2).

**Table 2:** Effect of ellagic acid on IPEC cell proliferation.

**Concentration μg/ml**	**Absorbance (OD value)**		
	**6 h**	**12 h**	**24 h**
0	0.594 ± 0.112	0.638 ± 0.03	0.768 ± 0.013
10	0.591 ± 0.033	0.748 ± 0.082	0.824 ± 0.062*
20	0.62 ± 0.086	0.807 ± 0.054**	0.937 ± 0.074^**#^
50	0.648 ± 0.06	0.94 ± 0.021^**##^	1.074 ± 0.021^**##^
100	0.695 ± 0.006^*#^	1.033 ± 0.06^**##^	1.096 ± 0.011^**##^

Compared with the control group, * means significant difference (P < 0.05), ** means extremely significant difference (P < 0.01); compared with the concentration is 10 μg / ml group, # means significant difference (P < 0.05), ## means extremely significant difference (P < 0.01).

#### Effect of LPS on the viability of IPEC-J2 cells

Following treatment with LPS for 12 h, IPEC-J2 cells treated with 10 μg/mL EA showed significantly lower proliferation than cells treated with 0 μg/mL (control group; *P* < 0.05). Following treatment with LPS for 24 h, IPEC-J2 cells treated with 1 and 10 μg/mL EA showed significantly lower proliferation than cells in the control group (*P* < 0.01). Following treatment with LPS for 48 h, IPEC-J2 cells treated with 0.1, 1, and 10 μg/mL EA showed significantly lower proliferation than cells in the control group (*P* < 0.01) (Table 3).

**Table 3:** Effect of LPS on IPEC cell proliferation.

**Concentration μg/ml**	**Absorbance (OD value)**		
	**12 h**	**24 h**	**48 h**
0	0.613 ± 0.109	0.764 ± 0.073	0.765 ± 0.109
0.1	0.583 ± 0.008	0.699 ± 0.055*	0.634 ± 0.053*
1	0.482 ± 0.084	0.602 ± 0.068**	0.526 ± 0.111**
10	0.336 ± 0.117*	0.363 ± 0.052**	0.352 ± 0.056**

Compared with the control group, * means significant difference (P < 0.05), ** means extremely significant difference (P < 0.01).

#### Effect of EA on the viability of LPS-induced IPEC-J2 cells

**Figure 9:**
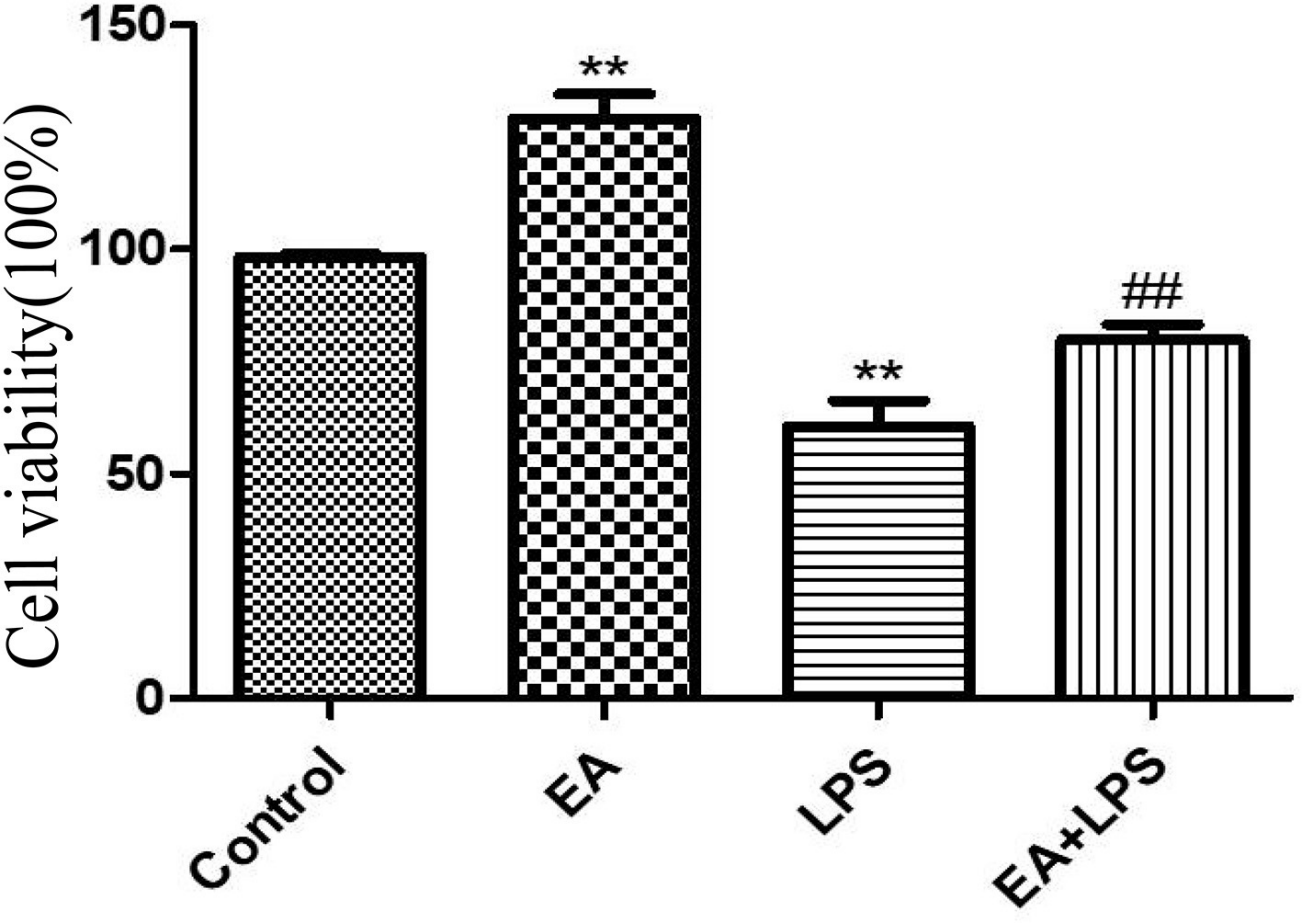
The effect of ellagic acid on LPS-induced IPEC-J2 inflammatory cell viability. Compared with the control group, ** means extremely significant difference (*P* < 0.01); compared with the LPS group, ## means extremely significant difference (*P* < 0.01).

LPS significantly reduced the viability of IPEC-J2 cells (*P* < 0.01), and EA significantly enhanced it (*P* < 0.01). In addition, EA significantly alleviated the inhibitory effects of LPS on the viability of IPEC-J2 cells (*P* < 0.01) (Figure 9). These results show that EA could reduce the harmful effects of LPS on IPEC-J2 cells.

#### Effect of EA on the MRNA expression of tight junction-related genes (ZO-1 and Occludin) in IPEC-J2 cells

**Figure 10:**
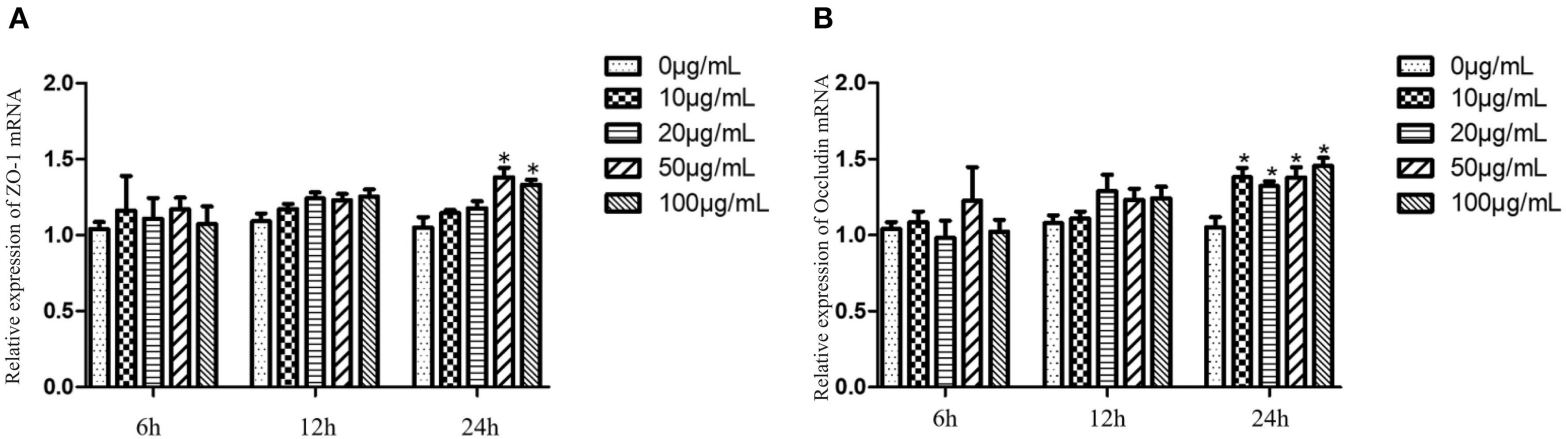
*ZO-1* and *Occludin* expression levels of IPEC-J2 cells treated with different concentrations of EA. **(A)** The expression levels of *ZO-1*; **(B)** the expression levels of *Occludin*. Compared with the control group, * means significant difference (*P* < 0.05).

The relative expression levels of *ZO-1* and *Occludin* in cells treated with EA for 6 and 12 h were not significantly different from those in the control group (*P* > 0.05). After treatment with EA for 24 h, the relative expression of *ZO-1* gradually increased in each treated group, and the levels were significantly higher in groups treated with 50 and 100 μg/mL EA than in the control group (*P* < 0.05). Of these treatment groups, the groups treated with 50 μg/mL EA had the highest expression levels of *ZO-1* (Figure 10A). At 24 h, the expression levels of *Occludin* were significantly higher in the EA-treated groups than in the control group (*P* < 0.05) (Figure 10B).

#### Effect of EA on the expression of inflammatory factors in LPS-induced IPEC-J2 cells

**Figure 11:**
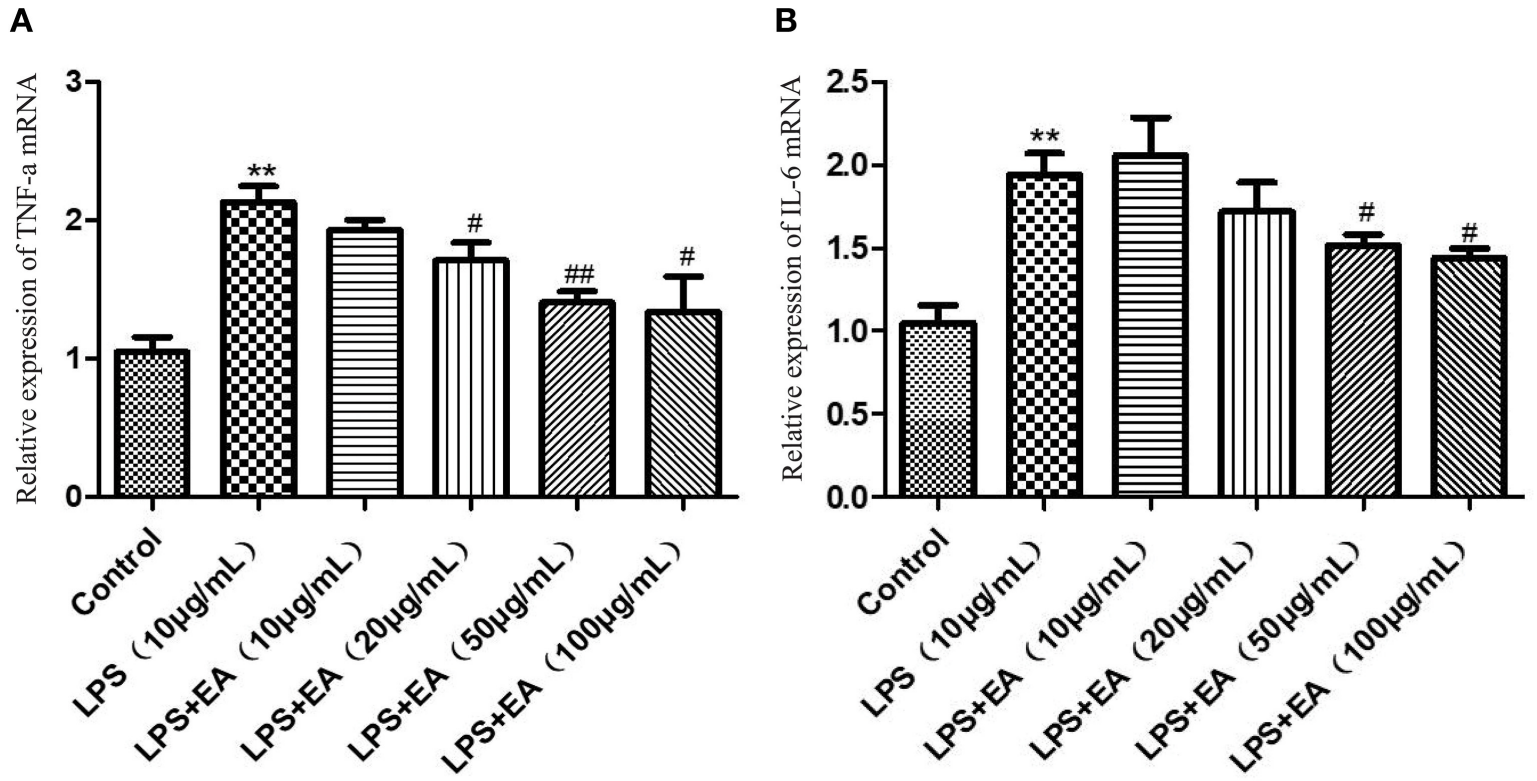
The expression levels of *TNF-*α and *IL-6* mRNAs in LPS-induced IPEC-J2 cells treated with different concentrations of EA. **(A)** The expression levels of *TNF-*α mRNA; **(B)** the expression levels of *IL-6* mRNA. Compared with the control group, ** means extremely significant difference (*P* < 0.01); compared with the LPS group, # means significant difference (*P* < 0.05), ## means extremely significant difference (*P* < 0.01).

The expression levels of *TNF-*α and *IL-6* mRNAs were significantly higher in the 10 μg/mL LPS group than in the control group (*P* < 0.01). Following this, EA solutions (10–100 μg/mL) were added to the 10 μg/mL LPS treatment group. The expression of *TNF-*α mRNA showed a downward trend. The down-regulation was significant at EA concentrations of 20 and 100 μg/mL (*P* < 0.05) and was extremely significant at an EA concentration of 50 μg/mL (*P* < 0.01) (Figure 11A). The *IL-6* mRNA expression levels also showed a downward trend following the addition of EA, and the decrease was significant at EA concentrations of 50 and 100 μg/mL (*P* < 0.05) (Figure 11B).

## Discussion

EA has numerous biological functions, including antioxidant, anti-cancer, and anti-inflammatory effects. Therefore, the practical applications of EA have received considerable research interest (11). DAO activity in peripheral blood is an important marker of damage to the mechanical barrier function of intestinal epithelial cells. In a previous study, we showed that DAO levels were significantly higher in the control group than the EA treated group (10), indicating that the piglets in the control group had suffered serious mechanical damage or inflammation during growth. The villous height in the small intestine was lower in the control group than in the test group, and some villi in the control group had been shed or damaged (Supplementary Table 5) (10). Taken together, these results suggested that EA may play a positive role in the repair of intestinal injury, the reduction of intestinal inflammation, and the growth of intestinal cells. Overall, these changes improved the nutritional absorption of piglets and promoted their growth and development.

In this study, we used high-throughput RNA-Seq technology to sequence genes and analyze their differential expression in the jejunal mucosa of experimental pigs fed different diets. We identified 401 differentially expressed genes and seven immune response pathways associated with the addition of EA in the diet. We screened key candidate genes from these pathways, including *CCL20, CCL5, CXCL11, CXCL16, IL2RB, IL2RG, IL9R, CD5, CD3E, CD244, TMIGD2, LAT, LCK*, and *ACKR3*. CC chemokines can activate specific chemokine receptors and cause inflammatory and non-inflammatory cells to migrate to sites of infection or damage, leading to a series of physiological and biochemical reactions. During inflammation, increased *CCL20* expression drives the recruitment of CCR6+ immune cells to regulate the immune response, inflammatory response, autoimmunity, and alloimmunity (12). *CCL5* recruits T cells and CCR5+ regulatory T cells (Tregs), which are immunosuppressive CD4+ T cells that control T cells, B cells, and NK cells, among others (13). CXC chemokines act as chemotactic cytokines that regulate the directional migration of inflammatory cells, promote cell proliferation and adhesion, and participate in the immune response. *CXCL11*—also known as interferon-inducible T cell α chemoattractant (I-TAC) or interferon γ-inducible protein 9 (*IP*-9)—also stimulates immune cells (14). *CXCL16* is known to regulate inflammation (15). *IL2RB* and *IL2RG* are interleukin 2 receptors β and γ. *IL*2*RB* can alter gene expression and signal transduction in T and NK cells as a result of early-onset autoimmunity and immunodeficiency (16). *IL9R* is a receptor of interleukin 9 (*IL*-9), which participates in various models of T cell-dependent inflammation and plays an important role in driving the immune responses to autoimmune diseases and chronic inflammation on mucosal surfaces (17, 18).

*CD5* and *CD3E* belong to the clusters of differentiation (CD) molecular family. The increased expression of *CD5* on T cells and B cells can prevent autoimmunity, and *CD3E* is positively associated with some T cells and macrophages (19, 20). *CD244* is a member of the signal lymphocyte activation molecule (SLAM) family. It is present in a variety of immune cells and plays an important regulatory role in immune and inflammatory responses (21). Granzyme B (*GZMB*) is a serine protease involved in inflammatory responses and autoimmunity (22). As a component of cytolytic granules in NK cells, it also participates in some pathological processes (23). *TMIGD*2 belongs to the Ig superfamily and is widely expressed on naive T cells, B cells, dendritic cells, and monocytes (24, 25). Linker for activation of T cells (*LAT*) and lymphocyte-specific protein tyrosine kinase (*LCK*) are key regulators of T cell development and function (26, 27). *ACKR*3 is an atypical chemokine receptor associated with some autoimmune diseases and inflammatory responses, and the *CXCL12*/*CXCR4*/*ACKR3* axis is a potential therapeutic target for several inflammatory diseases (28).

The large and diverse intestinal microbiota plays an important role in the growth and health of animals. Piglets can form a relatively stable microbiota structure in a short time after birth (29). Herein, we used 16S rDNA high-throughput sequencing to examine how dietary supplementation with EA influences the intestinal microbiota composition of piglets, and used PICRUSt analysis to predict the functions of gut bacterial microbiota. *Lactobacillus* were the most abundant and dominant group in the cecum and rectum of piglets fed an EA-supplemented diet. Previous studies have reported that *Lactobacillus* are beneficial bacteria that act as prebiotics and improve the intestinal barrier function, maintain gut homeostasis, and modulate immune responses in animals. The main *Lactobacillus* species in the intestine included *L. reuteri, L. salivarius, L. acidophilus, L. brevi*s, and *L. cellobiosas* (30, 31). We found that *L. delbrueckii* was the dominant microbe in the piglet cecum and may play a key role in pig intestinal health. A previous study has shown that *L. delbrueckii* effectively improves intestinal morphology, barrier function, immune response, and antioxidant capacity in LPS-challenged piglets (32). *Lactobacillus delbrueckii* also effectively enhances Tregs and reduces inflammatory cytokines and disease severity in systemic lupus erythematosus-induced mice (33). Herein, we found that *L. delbrueckii* promoted chemokines and inflammatory cytokines such as *CX3CL1, MIP3* α, *IL-6, IL-12*, and *IL-1* β in the piglet intestinal mucosa, thus causing physiological inflammation and promoting immune function (34). Dietary supplementation with *Lactobacillus delbrueckii* and *Lactobacillus salivarius* improved the level of growth hormone, immunity, and antioxidant functions in piglets, which helped reduce stress and improved their growth performance. This modulation of body growth could be attributed to the optimization of the gut bacterial community composition, mainly through the promotion of functional bacteria such as *Lactobacillus, L. delbrueckii, L. reuteri* and *L. salivarius* (35). Consistent with this result, we found that EA supplementation in the diet increased the abundance of *L. delbrueckii* in the cecum of piglets. Based on this, we speculate that *L. delbrueckii* may be the main target microbe of EA. Thus, EA supplementation results in *L. delbrueckii* becoming the dominant microbe in the intestine of piglets, which affects the intestinal immune response and promotes piglet growth.

*Lactobacillus reuteri* was the dominant intestinal microbe in the rectum of piglets. *Lactobacillus reuteri* shows good tolerance for the acidic environment of the gastrointestinal tract and can be added to the feed as a micro-ecological preparation. The main functions of *L. reuteri* included regulating the balance of the intestinal microbiota, promoting animal growth and development, improving feed utilization efficiency, inhibiting the activity of pathogenic microorganisms in the intestine, and promoting animal immunity (36). Karimi et al. reported that *L. reuteri* inhibits inflammatory responses in the mouse trachea by regulating T cell activity (37). Moreover, *L. reuteri* can help regulate the gut microbiota, relieve intestinal colitis, eliminate infections, and alleviate irritable bowel syndrome, antibiotic-related diarrhea, inflammatory bowel disease, and chronic constipation (38). Yi et al. reported that adding *L. reuteri* to the diet of weaned piglets increased the height of the mucosa in the ileum and reduced the depth of the duodenum. Simultaneously, the *ZO-1* and *Occludin* genes were highly expressed in the ileum, and the intestinal structure was more complete (39). Yang et al. reported that adding *L. reuteri* to the diet of newborn piglets increased the expression levels of *Claudin*-*1, ZO-1*, and *Occludin*, and protected tight junction protein-related genes in the LPS-induced IPEC-J2 inflammation model (40). Based on the results of previous studies, we speculated that EA supplementation would change the abundance of the dominant microbes in the intestine and cause *L. reuteri* to become a dominant microbe. Because *L. reuteri* plays a key role in intestinal immunity and intestinal tight junctions in animals, this change would improve immunity and anti-inflammatory functions in piglets fed an EA-supplemented diet.

The results of PICRUSt analysis indicated that the PTS function of the intestinal microbiota was enhanced in the test group. *Escherichia coli* primarily converts carbohydrates into shikimic acid and artemisinin *via* the PTS system (41). Shikimic acid has antibacterial, anti-tumor, anti-cerebral ischemia, anti-inflammatory, and analgesic effects (42, 43). LEfSe analysis revealed that Gram negative bacilli were more abundant in the rectum of piglets in the test group than in the control group. Therefore, we speculate that EA promotes the production of shikimic acid in the rectum to achieve anti-inflammatory and other effects.

IPEC-J2 plays an important role in the swine immune system and protects these animals from infectious and non-infectious environmental insults (44, 45). The tight junction structure in the intestinal tract plays a key role in regulating the impact of intestinal immune stress. While isolating the invasion of harmful factors, it allows small nutrient molecules to enter cells for the exchange of materials. If the tight junction structure is damaged, external harmful substances are no longer isolated by the mucosal barrier and can enter the intestinal tissue. This causes stress and induces an inflammatory response in the intestines of organisms (46, 47). *ZO-1* and *Occludin* are major members of the claudin family and play important roles in maintaining the integrity of the intestinal mucosa. *ZO-1* is involved in the signal transduction pathways related to barrier function and immune regulation (48). Moreover, the Occludin protein is an essential component of membrane proteins, and its ring structure and transmembrane properties are related to intestinal permeability (49). In this study, we found that EA enhanced the expression of the *ZO-1* and *Occludin* genes. This indicates that EA may have a positive effect on intestinal permeability and effectively protect the intestinal mucosal barrier function.

LPS induces IPEC-J2 cells to produce inflammatory factors, and excessive inflammatory factors can accelerate cell apoptosis and cause cellular inflammation (50). Stimulation by LPS is the primary reason for tissue damage and organ failure in animals, and LPS promotes the excessive release of inflammatory factors and causes death in severe cases. Intestinal mononuclear phagocytes and endothelial cells activated by LPS release inflammatory mediators such as TNF-α, IL-1, IL-6, IL-8, and histamine. The excessive release of inflammatory factors such as TNF-α and *IL-6* can trigger an inflammatory response in the intestinal mucosa, resulting in intestinal mucosal damage (51). Here, we induced IPEC-J2 cells with LPS and constructed an inflammation model. We found that LPS significantly reduced the viability of IPEC-J2 cells and increased the expression of *TNF-*α and *IL*-6. However, EA supplementation in the diet reduced the expression of *TNF-*α and *IL*-6, indicating that EA can alleviate the extent of LPS-induced inflammation. This finding confirmed the reliability of the results of transcriptome analysis and 16S rDNA sequencing in this study.

A study using the cecal contents of weaned piglets to analyze the intestinal microbial composition found that EA increased the level of functional intestinal microbes (7), which is consistent with our results. However, there is a lack of studies relating to intestinal cells. Our *in vitro* cell experiment found for the first time that EA helped maintain the integrity and barrier function of the intestinal mucosa, and affected the immune response of weaned piglets, thereby promoting the growth and development of piglets after weaning. These findings may have practical applications in terms of feeding practices in the swine industry. We speculate that EA changes the composition of the gut microbiota and increases the reproduction of some beneficial bacteria, which causes the downregulation of genes related to intestinal immune response. However, how the intestinal microbiota affects the expression of genes related to the intestinal immune response and inflammation needs further study.

## Conclusion

Dietary EA supplementation significantly increased the ADG and reduced the diarrhea rate and serum DAO levels of weaned piglets. Transcriptome sequencing of the jejunal mucosa of pigs fed an EA-supplemented diet showed that the significantly differentially expressed genes were mainly enriched in pathways related to immune response. Moreover, 16S rDNA sequencing of the cecal and rectal contents showed that the addition of EA influenced the bacterial microbiota composition in the intestine of piglets. This dietary treatment promoted microbes such as *Lactobacillus, L. delbrueckii*, and *L. reuteri*, thereby regulating the intestinal immune response. The results of *in vitro* experiments further showed that EA could effectively protect intestinal mucosal integrity and reduce the degree of LPS-induced inflammatory response. Overall, the results of this study indicate that dietary supplementation with EA can affect the intestinal mucosal immune response, maintain the intestinal health, and promote the growth performance of weaned piglets.

## Data availability statement

The datasets presented in this study can be found in online repositories. The names of the repository/repositories and accession number(s) can be found in the article/Supplementary material.

## Ethics statement

The animal study was reviewed and approved by the College of Animal Science and Technology, Guangxi University.

## Author contributions

YL and MZ undertook the analysis and wrote the paper. JM collated the data and wrote the paper. GL and JL reviewed and edited the manuscript. All authors have read and agree to the published version of the manuscript.

## Funding

This work was supported by grants from the National Natural Science Foundation of China (81860150), Special Project on Innovation Driven Development of Guangxi (Guike-AA18118051), and National Modern Agricultural Industrial Technology System (nycytxgxcxtd-15-01).

## Conflict of interest

Author MZ was employed by Guangxi Guiken Jinmao Animal Husbandry Co., Ltd. The remaining authors declare that the research was conducted in the absence of any commercial or financial relationships that could be construed as a potential conflict of interest.

## Publisher's note

All claims expressed in this article are solely those of the authors and do not necessarily represent those of their affiliated organizations, or those of the publisher, the editors and the reviewers. Any product that may be evaluated in this article, or claim that may be made by its manufacturer, is not guaranteed or endorsed by the publisher.
