# Supplementary material for: Dietary supplementation ellagic acid on the growth, intestinal immune response, microbiota, and inflammation in weaned piglets
Source: Front Vet Sci. 2022 Sep 2;9:980271. doi: 10.3389/fvets.2022.980271 (PMC9478910; doi:10.3389/fvets.2022.980271)
Supplement: Supplementary file 1 [file Table_1.docx]

Table S1 Basic diet formula and nutrition level

| Material | Content | Nutrient | Content |
| --- | --- | --- | --- |
| Quality corn (%) | 58 | Crude protein (%) | 18 |
| Dehulled soybean meal (%) | 25 | digestible energy(MJ/Kg) | 3 |
| Whey powder (%) | 7 | Crude fiber (%) | 5.5 |
| Super fish meal (%) | 3 | Calcium (%) | 0.6 |
| Calcium hydrogen phosphate (%) | 0.5 | Available phosphorus (%) | 0.25 |
| Sodium chloride (%) | 0.3 |  |  |
| Calcium carbonate (%) | 0.5 |  |  |
| Lysine (%) | 0.3 |  |  |
| Methionine (%) | 0.1 |  |  |
| Threonine (%) | 0.1 |  |  |
| Glucose (%) | 1.5 |  |  |
| White sugar (%) | 1.5 |  |  |
| Soybean oil (%) | 1 |  |  |
| Stone powder (%) | 0.2 |  |  |
| Premix (%) | 1 |  |  |
| Total (%) | 100 |  |  |

The premix provided the following per kilogram of complete diet: 110 mg of Fe as ferrous sulfate; 110 mg of Zn as zinc sulfate; 100 mg of Cu as copper sulfate; 30 mg of Mn as manganous oxide; 0.3 mg of Se as sodium selenite; 0.5 mg of I as ethylenediamine dihydroiodide; 10 000 IU of vitamin A as vitamin A acetate; 500 IU of vitamin D3; 100 IU of vitamin E; 2.0 mg of vitamin K3; 0.25 mg of biotin; 1.5 mg of folic acid; 15 mg of d-pantothenic acid; 45 µg of vitamin B12; 7 mg of vitamin B2; 2 mg of vitamin B1; 5 mg of vitamin B6.

Table S2 The primer sequence of RT-qPCR

| Items | Gene | Forward | Reverse |
| --- | --- | --- | --- |
| Target | GCG | 5'-TCATTCCCAGCTCCCCAGACGGAC-3' | 5'-TCATTCCCAGCTCCCCAGACGGAC-3' |
|  | ACOX1 | 5'-CCTGATCGAAGCCTATAGACTG-3' | 5'-CTGAATCTGGTGGAGTTTTTCG-3' |
|  | ECH1 | 5'-GCACCTCCATAATCTCATCTCC-3' | 5'-GGCTCTGGTTTCCAATGATTTT-3' |
|  | GIP | 5'-GCGACTGGAAACACAACATCACCC-3' | 5'-AGCAGCTCTCGAGTCAGTACATCC-3' |
|  | PYY | 5'-AACCTGGTCACTCGGCAGAGGTA-3' | 5'-GGGATCGCGAGCAAACATGCAAA-3' |
|  | SLC5A10 | 5'-CACCATGCCGGAGTACATCCAG-3' | 5'-TTCCAGCCCAGACAGATGTGC-3' |
|  | SLC25A24 | 5'-GAGTTGATTCTCCGAAGCATTG-3' | 5'-TCATGACTTTCAGACGATCCAA-3' |
| Reference | 18S | 5'-CCCACGGAATCGAGAAAGAG-3' | 5'-TTGACGGAAGGGCACCA-3' |

Table S3 The tight junction-related genes primer sequence

| Items | Gene | Forward | Reverse |
| --- | --- | --- | --- |
| Target | TNF-a | 5'-TCTCCTTCCTCCTGGTCGCA-3' | 5'-TCCCTCGGCTTTGACATTGG-3' |
|  | IL-6 | 5'-AGCCCACCAGGAACGAAAGA-3' | 5'-AGCCATCACCAGAAGCAGCC-3' |
|  | Zo-1 | 5'-GATCCTGACCCGGTGTCTGA-3' | 5'-TTGGTGGGTTTGGTGGGTTG-3' |
|  | Occludin | 5'-ATCAACAAAGGCAACTCT-3' | 5'-GCAGCAGCCATGTACTCT-3' |
| Reference | 18S | 5'-CCCACGGAATCGAGAAAGAG-3' | 5'-TTGACGGAAGGGCACCA-3' |

Table S4. Growth performance, Diarrhea rate and Dao activity of test group (10)

| Item | Determination result | | *P*-value |
| --- | --- | --- | --- |
|  | Control group | Test group |  |
| Initial weight/kg | 9. 61 ± 0. 98 | 9. 66 ± 1. 41 | 0. 930 |
| Final weight /kg | 26. 10 ± 2. 85 | 28. 44* ± 1. 96 | 0. 045 |
| Average daily gain /(kg·d－1 ) | 0. 416 ± 0. 068 | 0. 489* ± 0. 062 | 0. 042 |
| Diarrhea rate/% | 4. 83 ± 0. 09 | 3. 17* ± 0. 06 | 0. 046 |
| DAO/( U·mL^－1^ ) | 6. 88 ± 1. 09 | 5. 24* ± 0. 59 | 0. 049 |

Compared with the control group, * means significant difference (*P* < 0.05)

Table S5. The results of intestinal morphology of weaned piglets (10)

| Item | Group | Villus height/μm | Crypt depth/μm | Villus height/Crypt depth |
| --- | --- | --- | --- | --- |
| Jejunum | control | 302. 87 ± 36. 6 | 164. 45 ± 21. 76 | 1. 86 ± 0. 24 |
|  | test | 348. 39* ± 30. 49 | 127. 46* ± 17. 02 | 2. 74* ± 0. 47 |
| Ileum | control | 266. 21 ± 21. 90 | 161. 66 ± 11. 30 | 1. 65 ± 0. 08 |
|  | test | 305. 59* ± 22. 05 | 141. 32 ± 20. 10 | 2. 18* ± 0. 29 |

Compared with the control group, * means significant difference (*P* < 0.05)
